# *Pelagia noctiluca* (Scyphozoa) Crude Venom Injection Elicits Oxidative Stress and Inflammatory Response in Rats

**DOI:** 10.3390/md12042182

**Published:** 2014-04-10

**Authors:** Giuseppe Bruschetta, Daniela Impellizzeri, Rossana Morabito, Angela Marino, Akbar Ahmad, Nunziacarla Spanò, Giuseppa La Spada, Salvatore Cuzzocrea, Emanuela Esposito

**Affiliations:** 1 Department of Biological and Environmental Sciences, University of Messina, Viale Ferdinando Stagno D’Alcontres 31, Messina 98166, Italy; E-Mails: bruschettag@unime.it (G.B.); dimpellizzeri@unime.it (D.I.); marinoa@unime.it (A.M.); aahmad@unime.it (A.A.); nunziacarla.spano@unime.it (N.S.); salvator@unime.it (S.C.); eesposito@unime.it (E.E.); 2 Department of Human and Social Sciences, University of Messina, Via T. Cannizzaro 278, Messina 98122, Italy; E-Mail: rmorabito@unime.it; 3 Manchester Biomedical Research Centre, Manchester Royal Infirmary, University of Manchester, Manchester M13 9PL, UK

**Keywords:** crude venom, *Pelagia noctiluca*, oxidative stress, inflammation, apoptosis, tempol

## Abstract

Cnidarian toxins represent a rich source of biologically active compounds. Since they may act via oxidative stress events, the aim of the present study was to verify whether crude venom, extracted from the jellyfish *Pelagia noctiluca*, elicits inflammation and oxidative stress processes, known to be mediated by Reactive Oxygen Species (ROS) production, in rats. In a first set of experiments, the animals were injected with crude venom (at three different doses 6, 30 and 60 µg/kg, suspended in saline solution, i.v.) to test the mortality and possible blood pressure changes. In a second set of experiments, to confirm that *Pelagia noctiluca* crude venom enhances ROS formation and may contribute to the pathophysiology of inflammation, crude venom-injected animals (30 µg/kg) were also treated with tempol, a powerful antioxidant (100 mg/kg i.p., 30 and 60 min after crude venom). Administration of tempol after crude venom challenge, caused a significant reduction of each parameter related to inflammation. The potential effect of *Pelagia noctiluca* crude venom in the systemic inflammation process has been here demonstrated, adding novel information about its biological activity.

## 1. Introduction

Plant- and animal-derived drugs, whose discovery and biological activity have been under investigation for years, are reported as a good research model for studying protein structure-function relationship, ascertaining biological mechanisms and as potential candidates for drug development [1,2]. Among venomous marine animals, the ancient *phylum* of Cnidaria includes Anthozoa (sea anemones and corals) and Medusozoa. This latter comprises Cubozoa, Hydrozoa, Scyphozoa (known as true jellyfish) and Staurozoa. All of them represent an interesting source of biologically active compounds [2,3,4,5,6,7]. This relevant aspect is due to their peculiar cells, called nematocytes, specialized stinging cells, located in tentacles and oral arms. They produce an organoid, the nematocyst, consisting of a capsule wall containing an inverted tubule and a fluid matrix in which various toxins are stored. Under both a chemical and mechanical stimulus, the tubule is rapidly ejected adhering to or penetrating the prey or aggressor, thus injecting toxins (discharge) [8]. Toxins delivery induces damage at different levels of the target organism: by affecting cellular membranes and ion fluxes or altering myocardium, or nervous tissue, hepatic tissue and kidney functions or provoking release of inflammatory mediators [2,3,4,9]. Such effects may be explained by the presence and mechanism of action of vasoactive amines, kinins, collagenases, proteases, fibrinolysins, dermoneurotoxins, cardiotoxins, neurotoxins, myotoxins and antigenic proteins that, at least in part, make up cnidarian toxins [7,10,11]. Several studies have been performed to characterize toxins from many poisonous animals such as cone-snails, scorpions, snakes and spiders [12,13], but cnidarian toxins need to be further focused on. This is surprising because, although most Cnidaria do not have harmful nematocysts that are able to penetrate human skin, the accidental contact with some cubozoans, such as *Chironex fleckeri* (the Pacific Sea Wasp), *Carukia barnesi* and *Malo kingi* (the latter two are both commonly called Irukandji Jellyfish) can be lethal [14]. Other medusozoan *taxa*, species of both planktonic [15] and benthic animals [16], are also involved in human envenomation.

Among Scyphozoa, *Pelagia noctiluca*, a holoplanktonic epipelagic specimen, is one of the most dangerous jellyfish in the Mediterranean Sea, where its blooming has been very abundant for many years [10]. Both *in vivo* and *in vitro* biological assays have been performed to verify and, possibly, measure the toxicity of *Pelagia noctiluca* crude venom, whose composition has still not been completely defined [6,17,18]. The accidental contact of humans with this jellyfish induces the simultaneous discharge of nematocysts, injecting the venom and causing local pain, burning, swelling, hyper pigmentation and other local symptoms [10]. A recent study demonstrated that tentacle extract from the jellyfish *Cyanea capillata* caused delayed jellyfish envenomation syndrome (DJES) with serious multiple organ dysfunction or systemic damages [19].

As a general feature, cnidarian toxins may cause damage through two mechanisms of action: they may create pores into the target membrane, provoking both ions and water unbalance, or may induce oxidative stress events, leading to lipoperoxidation [20,21,22].

Reactive oxygen species (ROS) play an important role in inflammation and shock: ROS (particularly hydroxyl radicals) may initiate cell membrane lipid peroxidation, react with proteins in the plasma to produce chemotactic agents and increase microvascular permeability. Shock and inflammation are associated with superoxide anion production and the simultaneous production of superoxide and NO promotes the generation of a highly cytotoxic reaction product, the oxidant peroxynitrite (ONOO^−^) [23,24,25,26]. In various forms of shock and inflammation, peroxynitrite has been proposed to significantly contribute to the cellular metabolic failure and vascular injury [27,28,29,30]. The hypothesis that ROS generation contributes to inflammation is supported by many studies, demonstrating the beneficial effects of various interventions, which either reduce ROS generation or reduce their effects. These therapeutic strategies include the administration of antioxidant enzymes, such as superoxide dismutase (SOD) and catalase (CAT), ROS scavengers, such as mannitol, and agents preventing ROS generation such as desferrioxamine (DEF) and allopurinol [31,32]. Thus, SOD mimetics, are potentially useful in conditions associated with inflammation [33]. In this regard, tempol (4-hydroxy-2,2,6,6-tetramethylpiperidine-*N*-oxyl), a member of a family of nitroxide compounds, has been extensively studied in animal models of increased ROS levels and in hypertension and endothelial function. The chemistry of tempol [34] and its effects on blood pressure (BP) regulation and endothelial function [35] have been already reviewed. The reaction of tempol with superoxide anion (O_2_^•−^) to form hydrogen peroxide (H_2_O_2_) accounts for its superoxide dismutase (SOD) mimetic effect [36]. Tempol also reduces hydroxyl radicals formation and the intracellular concentrations of Fe_21_ and, hence, the formation of hydroxyl radicals via the Fenton or Haber-Weiss reactions. An extensive series of studies [37] defined the biophysical properties of nitroxides and their roles as radiation protection agents. This led the way to the first use of tempol in human subjects where it is undergoing clinical trial as a topical agent to protect hair and skin of patients receiving cranial radiotherapy for cancer [38].

Based on these observations, in this study we aimed to investigate the systemic inflammation and oxidative stress elicited by crude venom extracted from nematocysts of *Pelagia noctiluca* and, consequently, the possible effect of a powerful antioxidant, such as tempol, on the reduction of crude venom induced inflammatory response.

Specifically, we investigated the effect of tempol on mortality; lung and intestine injury (histology); formation of nitrotyrosine, intercellular adhesion molecule-1 (ICAM-1), P-Selectin, inducible nitric oxide synthase (iNOS) (immunohistochemistry); poly(adenosine 59-diphosphate-ribose) synthetase (PARS) activity.

## 2. Results

### 2.1. Effect of Pelagia noctiluca Crude Venom on Blood Pressure and Mortality

**Figure 1:**
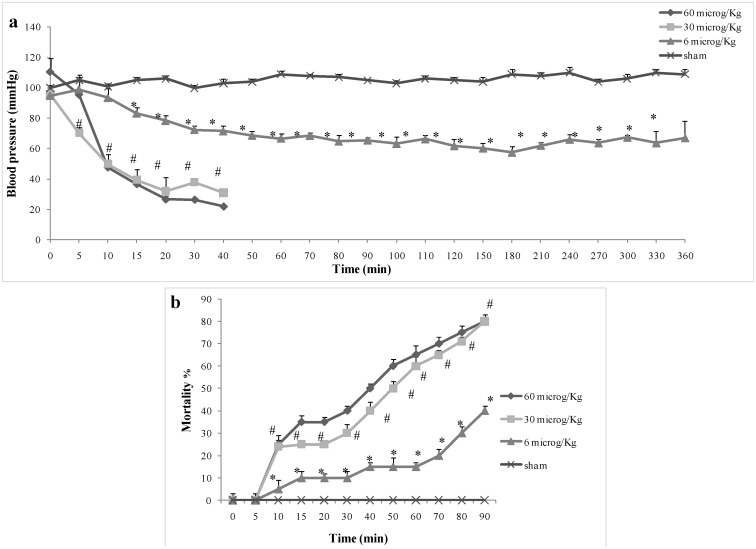
Effect of *Pelagia noctiluca* crude venom toxicity on blood pressure and mortality. A significant reduction of blood pressure (**a**) and increase of mortality (**b**) was observed at 30 and 60 µg/kg compared to 6 µg/kg dose within 1 h. No substantial difference between 30 and 60 µg/kg was found in crude venom injected rats. No alterations in blood pressure and mortality percent were found in the sham group (**a**,**b**). Data are means ± SEM of 25 rats for each group. Crude venom 60 µg/kg * *p* < 0.05 *vs.* 6 µg/kg crude venom, ^# ^*p* <0.05 *vs.* crude venom 30 µg/kg.

To better understand the inflammatory effects of *Pelagia noctiluca* crude venom in injected rats, we preliminary monitored blood pressure within 3 h and mortality within 90 min, at three different doses of crude venom (6, 30 and 60 µg/kg). A significant reduction of blood pressure and increase of mortality percent at 30 and 60 µg/kg compared to 6 µg/kg were observed (Figure 1a,b). No significant difference was found between the dose of 30 and 60 µg/kg (Figure 1a,b). No blood pressure alterations and no mortality percent was found in the sham group (Figure 1a,b). Based on these preliminary results, 30 µg/kg crude venom dose was considered for experiments. Thus, for the next step, to investigate the mechanisms related to the inflammatory process and oxidative stress possibly induced by *Pelagia noctiluca* crude venom, venom-injected rats were treated with tempol, as a potent antioxidant.

### 2.2. Effect of Crude Venom on Hepatocellular, Pancreatic and Renal Dysfunction

**Figure 2:**
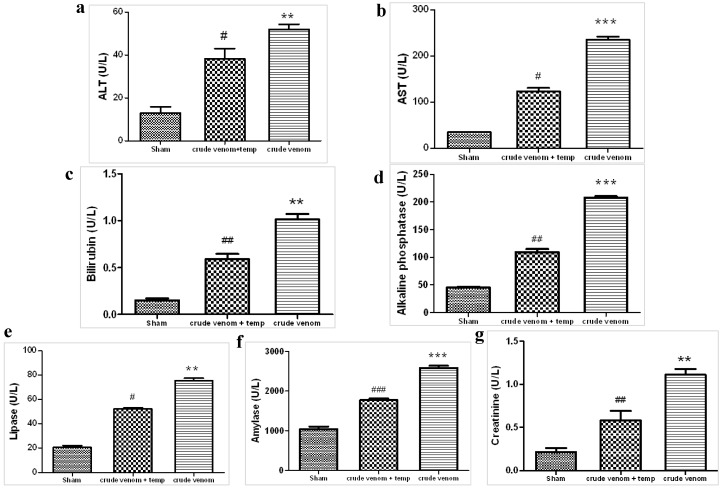
Effect of *Pelagia noctiluca* crude venom on hepatocellular, pancreatic and renal dysfunction. Crude venom-injected rats exhibit an increased plasma concentration of alanine aminotransferase (**a**), aspartate aminotransferase (**b**), bilirubin (**c**), alkaline phosphatase (**d**), lipase (**e**), amylase (**f**) and creatinine (**g**). Administration of tempol prevented these metabolic modifications (a–g). Data are means ± SEM of 25 rats for each group. ** *p* < 0.01 *vs.* sham, *** *p* < 0.001 *vs.* sham, ^# ^*p* < 0.05 *vs.* crude venom, ^## ^*p* < 0.01 *vs.* crude venom, ^### ^*p* < 0.001 *vs.* crude venom.

Hepatocellular injury*.* Crude venom injected rats significantly exhibited higher ALT (Figure 2b), AST (Figure 2a), bilirubin (Figure 2c) and alkaline phosphatase (Figure 2d) plasma levels, compared to the control group. All these findings are consistent with the development of hepatocellular injury. Treatment with tempol significantly decreased the liver injury caused by crude venom injection.

Pancreatic injury. Crude venom injected rats exhibited significantly higher lipase and amylase levels than the control group: such findings are consistent with the development of pancreatic injury (Figure 2e,f). Treatment with tempol significantly decreased the pancreatic injury caused by crude venom injection (Figure 2e,f).

Renal dysfunction. Crude venom injected rats exhibited a significant increase in plasmatic creatinine concentration compared to the control group, predictive of renal dysfunction development (Figure 2g). Treatment reduced the crude venom-induced renal dysfunction (Figure 2g).

### 2.3. Histological Evaluation after Pelagia noctiluca Crude Venom Treatment

**Figure 3:**
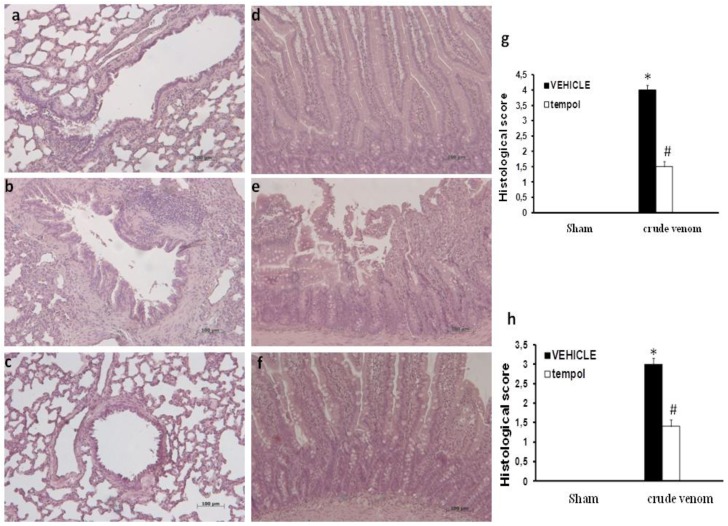
Effect of *Pelagia noctiluca* crude venom on histological evaluation of lung and intestine. Representative lung (**a**,**g**) and intestine (**d**,**h**) sections from sham rats demonstrate the normal architecture of these organs. On the contrary, lung and intestine biopsies showed marked inflammatory changes with pronounced cellular infiltration after crude venom administration (**b**,**e**). Treatment with tempol significantly reduced the pathological changes in the tissues (**c**,**f**). Figures are representative of at least three experiments performed on different experimental days. * *p* < 0.05 *vs.* sham, ^# ^*p* < 0.05 *vs.* crude venom.

No histological alteration was observed in tissues from control group rats (Figure 3a,d). Histological evaluation of lung (Figure 3b) and intestine sections (Figure 3e) 6 h after crude venom injection (30 µg/kg) revealed marked pathological changes. In particular, lung and intestine sections revealed inflammatory infiltration by neutrophils, macrophages and plasma cells (Figure 3b,e). In that regard, *Pelagia noctiluca* crude venom caused a significant histological damage in injected rats, the treatment with tempol caused a substantial reduction in the extent of histological damage in both lung and intestine (Figure 3c,f).

### 2.4. Effect of Pelagia noctiluca Crude Venom on the Degradation of IκB-α and Translocation of NF-kB p65

**Figure 4:**
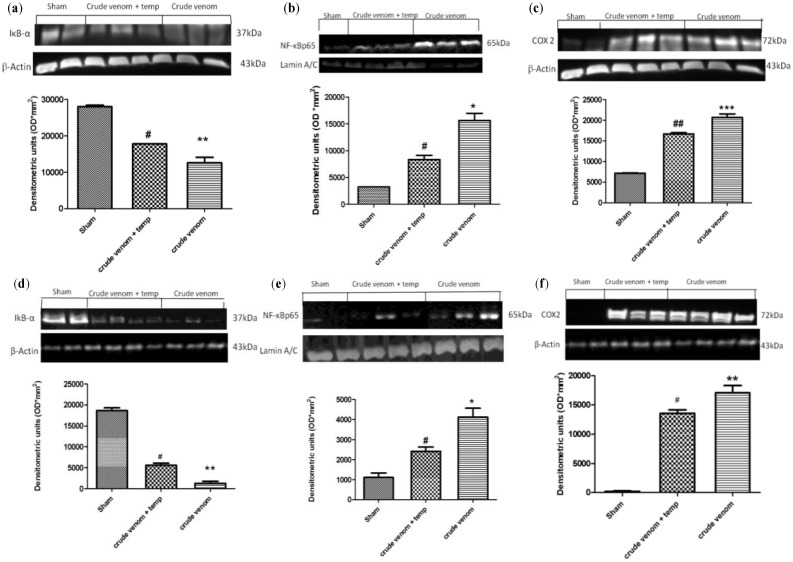
Effect of *Pelagia noctiluca* crude venom on inhibitor of kappa B (IκB-α), nuclear factor kappa B (NF-κB p65) and cyclooxygenase-2 (COX2) expression. A basal level of IκB-α was detected in lung (**a**) and intestine (**d**) tissue homogenates of control group animals. After crude venom administration, IκB-α levels were decreased, while in tempol-treated rats IκB-a levels were substantially increased. Moreover, NF-κB p-65 levels in lung (**b**) and intestine (**e**) tissues were significant increased by *Pelagia noctiluca* crude venom administration, compared with the control group rats. NF-κB p-65 levels were significantly reduced in the nuclear fractions of these organs from animals that had received tempol treatment. In addition, the expression of COX2 was increased in lung (**c**) and intestine (**f**). Tempol treatment significantly reduced COX2 levels in both organs. The results are expressed as mean ± SEM from *n* = 5/6 lungs and intestine tissues for each group. * *p* < 0.05 *vs.* sham, ** *p* < 0.01 *vs.* sham, *** *p* < 0.001 *vs.* sham, ^# ^*p* < 0.05 *vs.* crude venom, ^## ^*p* < 0.01 *vs.* crude venom. β-actin and laminin were used as internal control.

Inhibitor of kappa B (IκB-α) degradation and nuclear factor kappa B (NF-κB p65) expression were evaluated by Western blot analysis to investigate the cellular mechanisms underlying the development of acute lung and intestine injury after injection with crude venom. Basal expression of IκB-α was detected in lung (Figure 4a) and intestine samples (Figure 4d) from control group animals, whereas IκB-α levels were substantially reduced in both lung and intestine tissues obtained from venom-injected animals. Treatment with tempol prevented crude venom-induced IκB-α degradation, with increased IκB-α levels in treated rats (Figure 4a,d). Moreover, NF-κB p65 levels in lung (Figure 4b) and intestine (Figure 4e) nuclear fractions were also significantly increased at 6 h after crude venom injection, compared to control group rats. In that regard, we demonstrated that tempol treatment significantly reduced NF-κB p65 levels in both lung and intestine inflamed tissues.

### 2.5. Effect of Pelagia noctiluca Crude Venom on COX2 Expression

The expression of cyclooxygenase-2 (COX2) was also assessed by Western blot analysis, to better understand the effects of *Pelagia noctiluca* crude extract on lipid degradation processes and on the subsequent production of leukotrienes and prostaglandins. As seen in the figure, the expression of COX2 is increased in lung (Figure 4c) as well as in intestine (Figure 4f), compared to the control group. On the other hand, rats treated with tempol showed a significant reduction in COX2 levels in both organs (Figure 4c,f).

### 2.6. Effect of Pelagia noctiluca Crude Venom on P-Selectin and ICAM-1 Expression and MPO Activity

**Figure 5:**
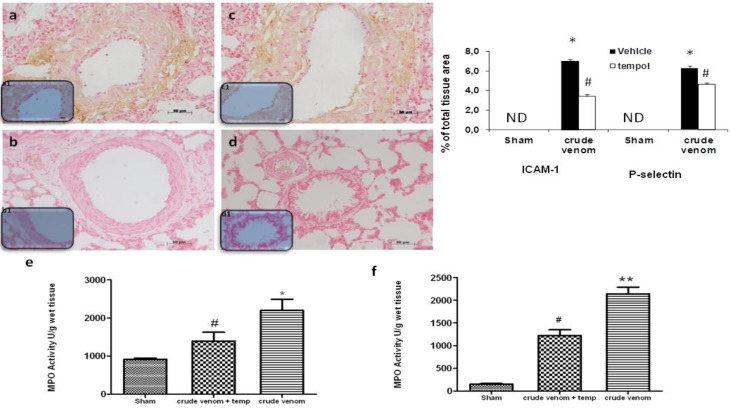
Effect of *Pelagia noctiluca* crude venom on the adhesion molecules expression and MPO activity. No positive staining for ICAM-1 and P-selectin was found in the lungs as well as in the intestine from control group rats. Six hours after crude venom injection, a positive ICAM-1 and P-selectin staining was found in lung (**a**,**c**; see particles **a1**,**c1**) and intestine (data not shown). There was no detectable immunostaining for ICAM-1 and P-Selectin in lungs and intestine (**b**,**d**; see particles **b1**,**d1**) of crude venom-injected rats treated with tempol. In addition, MPO activity was increased significantly in lung (**e**) and intestine (**f**) of the crude venom-treated rats, compared with sham rats. Tempol treatment significantly reduced the crude venom-induced increase in myeloperoxidase activity. Figures are representative of at least three experiments performed on different experimental days. Data are means ± SEM of 25 rats for each group, * *p* < 0.05 *vs.* sham, ** *p* < 0.01 *vs.* sham ^# ^*p* < 0.05 *vs.* crude venom.

Six hours after crude venom injection, expression of the adhesion molecules ICAM-1 and P-Selectin were evaluated to assess neutrophil infiltration. In crude venom-injected rats, an increase in immunohistochemical staining for ICAM-1 and P-Selectin was demonstrated on the surface of endothelial cells in both inflamed lung (Figure 5a; see particles a; Figure 5c; see particles c1) and intestine (data not shown), while the immunostainings for ICAM-1 and P-Selectin were markedly reduced in lung (Figure 5b,d; see particles b1,d1) and intestine (data not shown) tissues of tempol-treated rats. No staining for either ICAM-1 or P-Selectin was found in tissue sections obtained from the control group (data not shown). Six hours after crude venom injection, MPO activity, accounting for PMN infiltration, was measured in lung (Figure 5e) and intestine (Figure 5f). As shown in figure, MPO activity was significantly increased in both organs 6 h after crude venom injection. MPO activity was also significantly reduced by tempol treatment (Figure 5e,f).

### 2.7. Effects of Pelagia noctiluca Crude Venom on NO Production

**Figure 6:**
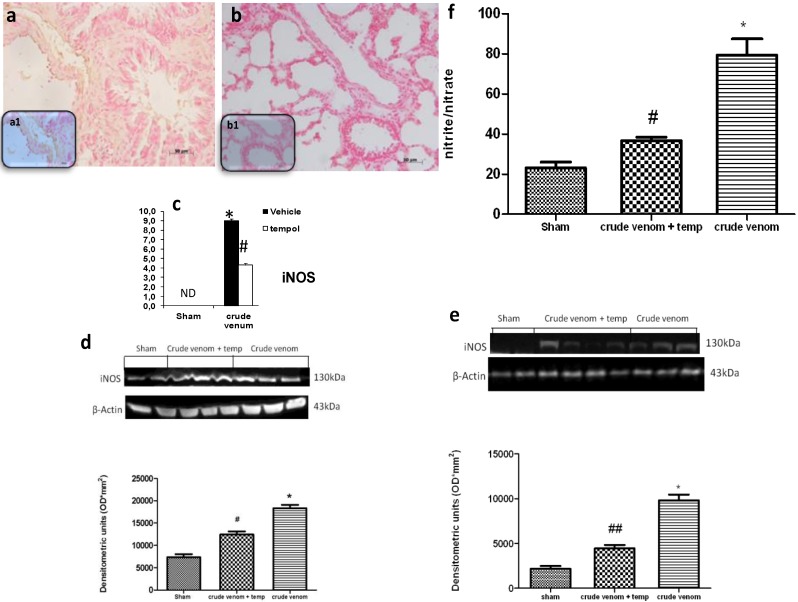
Effect of *Pelagia noctiluca* crude venom on nitric oxide (NO) production. Immunohistochemical localization of iNOS in lung tissue 6 h after *Pelagia noctiluca* crude venom injection showed a positive staining (**a**; see particles a1), compared with control group. The expression of iNOS was significantly attenuated in samples collected from rats that had received tempol treatment (**b**; see particles b1). In addition, iNOS expression and plasma levels of NO_2_^−^/NO_3_^−^ were also measured (**d**–**f**). A significant increase in the iNOS expression and plasma levels NO_2_^−^/NO_3_^−^ were observed in lung (**d**) and in intestine (**e**) of crude venom injected rats, compared to the control group. Tempol treatment significantly reduced both iNOS and NO_2_^−^/NO_3_^−^ levels (**f**). Densitometry analysis (**c**) of immunocytochemistry photographs (*n* = 5 photos from each sample collected from all rats in each experimental group) for iNOS from lung was assessed. The assay was carried out by using Optilab Graftek software on a Macintosh personal computer (CPU G3-266). Data are expressed as % of total tissue area. Figures are representative of at least three experiments performed on different experimental days. Data are means ± SEM of 25 rats for each group, * *p* < 0.05 *vs.* sham, ^# ^*p* < 0.05 *vs.* crude venom, ^## ^*p* < 0.01 *vs.* crude venom. β-actin was used as internal control.

To determine the role of nitric oxide (NO) produced by the injection of crude venom, iNOS expression was evaluated by Western blot and immunohistochemistry. Samples of lung tissue, taken 6 h after injection, were processed for immunohistological staining for iNOS. Lung sections from control group rats did not stain for iNOS (data not shown), whereas lung sections obtained from crude venom-treated rats exhibited a positive staining for iNOS (Figure 6a; see particles a1). Here, we also showed a reduction in NO production and iNOS activity in lung (Figure 6b; see particles b1) and intestine (data not shown) by tempol treatment. Western blot analysis revealed a markedly reduced iNOS expression in lung (Figure 6d) and intestine (Figure 6e) tissues from tempol-treated rats. Both groups showed a significant increase of expression compared to the control group. In addition, plasma analysis showed that the injection of crude extract of *Pelagia noctiluca* induced a significant concentration of NOx compared to sham rats. The increase in plasma NO_2_/NO_3_ levels was significantly reduced in tempol-treated animals (Figure 6f).

### 2.8. Effect of Pelagia noctiluca Crude Venom on Nitrotyrosine Production, PARP Activation and malondialdehyde (MDA) Levels

**Figure 7:**
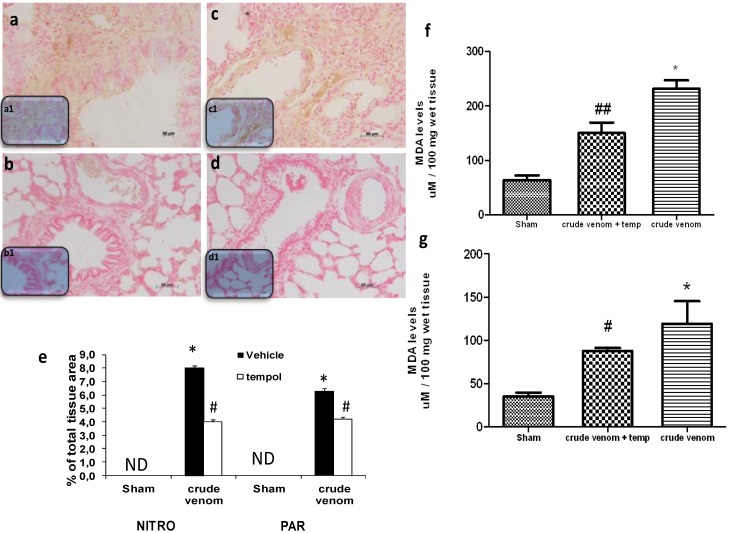
Effect of *Pelagia noctiluca* crude venom on nitrotyrosine production, Poly ADP-ribose (PAR) activation and malondialdehyde (MDA) levels. There was no staining for nitrotyrosine and PAR in lung tissues from sham rats. A positive nitrotyrosine and PAR staining was found in lung tissues collected after crude venom administration (**a**,**c**; see particles a1,c1). Nitrotyrosine and PAR expression were significantly attenuated in lung tissues collected from crude venom injected rats treated with tempol (**b**,**d**; see particles b1,d1). In addition, an increase of lipoperoxidation degree was found in lung (**f**) and in intestine (**g**) collected after crude venom administration, compared with sham animals. Tempol treatment reduced these organs MDA levels. Densitometry analysis (**e**) of immunocytochemistry photographs (*n* = 5 photos from each sample collected from all rats in each experimental group). The assay was carried out by using Optilab Graftek software on a Macintosh personal computer (CPU G3-266). Figures are representative of at least three experiments performed on different experimental days. Data are means ± SEM of 25 rats for each group, * *p* < 0.05 *vs.* sham, ^# ^*p* < 0.05 *vs.* crude venom ^## ^*p* < 0.01 *vs.* crude venom.

Six hours after crude venom injection, the presence of nitrotyrosine was investigated in both lung and intestine sections. Immunohistochemical analysis, using a specific anti-nitrotyrosine antibody, revealed a positive staining in lung (Figure 7a; see particles a1) and intestine (data not shown) of crude venom-injected rats. Treatment with tempol reduced nitrotyrosine staining in both organs from crude venom-challenged rats (Figure 7b; see particles b1). Immunohistochemical analysis of lung (Figure 7c; see particles c1) and intestine (data not shown) sections, obtained from crude venom-challenged rats, also revealed a positive staining for Poly ADP-ribose (PAR). In contrast, staining for PAR was absent in sections of lung (Figure 7d; see particles d1) and intestine (data not shown) from tempol-treated crude venom-challenged rats. There was no staining for either nitrotyrosine or PAR in sections of lung (data not shown) or intestine (data not shown) from control group rats. In addition, 6 h after crude venom injection, MDA concentration was measured as an indicator of the degree of lipid peroxidation. As shown in figure, MDA levels were significantly increased in lung (Figure 7f) and intestine (Figure 7g), while MDA levels were significantly reduced by tempol treatment (Figure 7f,g).

### 2.9. Effects of Pelagia noctiluca Crude Venom on Apoptotic Proteins Expression

**Figure 8:**
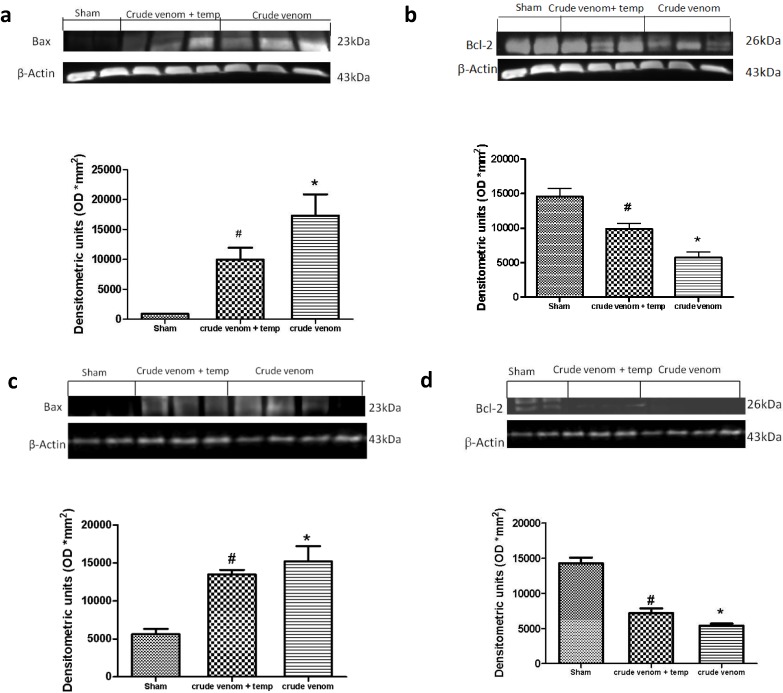
Effect of *Pelagia noctiluca* crude venom on the expression of Bax and Bcl-2. Western blot analysis of Bax and Bcl-2 levels was performed in lung (**a**,**c**) and intestine (**b**,**d**) samples 6 h after *Pelagia noctiluca* crude venom-injection. A significant increase in Bax expression was observed in these organs (**a**,**c**) compared with the sham-treated animals, whereas in tempol-treated rats Bax levels were substantially reduced. High expression of Bcl-2 was found in sham animals. On the contrary, 6 h after *Pelagia noctiluca* crude venom administration, a decrease in the Bcl-2 expression was observed (**b**,**d**) compared with the control group. Bcl-2 expression was more evident in lung and intestine tissue from *Pelagia noctiluca* crude venom-treated rats that received tempol treatment. Data are means ± SEM of 25 rats for each group, * *p* < 0.05 *vs.* sham, ** *p* < 0.01 *vs.* sham, ^# ^*p* < 0.05 *vs.* crude venom. β-actin was used as internal control.

Six hours after crude venom injection, the appearance of proapoptic protein Bax in lung (Figure 8a) and intestine (Figure 8c) homogenates was investigated by Western blot, showing that Bax levels were appreciably increased (Figure 8a,c). In addition, Bcl-2 expression was analyzed by Western blot analysis in homogenates from lung (Figure 8b) and intestine (Figure 8d) tissues. A basal level of Bcl-2 expression was detected in tissues of the control group (Figure 8b,d), while 6 h after crude venom injection, Bcl-2 expression was significantly reduced in lung and intestine (Figure 8b,d). Treatment with tempol induced a decrease of Bax (Figure 8a,c) and an increase in Bcl-2 expression (Figure 8b,d) in both lung and intestine tissues from injected animals, attenuating the effect of *Pelagia noctiluca* crude venom on the apoptotic pathway.

## 3. Discussion

Jellyfish envenomation in some cases may result in rapid death of animals or human victims (5–20 min), usually due to cardiorespiratory arrest [39], which arouses researchers’ great interest. However, death due to jellyfish sting is very rare. Most victims show dermatological symptoms, and only a small proportion require hospitalization for further treatment [40]. It is rather difficult to perform a unitary dissertation about toxicological features of Cnidaria, due to the variety of specimens and to the different techniques employed to obtain the venom. In spite of many data about Cnidaria toxins [41,42,43], toxicological features of *Pelagia noctiluca* are still not completely understood. One of the most relevant aspects of *Pelagia noctiluca* is related to the biologically active compounds contained inside its nematocysts [6,17,18,44]. The accidental contact with the jellyfish *Pelagia noctiluca* can actually produce severe local and systemic pathologies, including inflammatory events [10]. With regard to the latter point, there is a large body of evidence showing that the production of reactive oxygen and nitrogen species play key roles in inflammation caused by *Pelagia noctiluca* crude venom [20,45]. In this respect, the present experiments demonstrate that *Pelagia noctiluca* venom caused a significant reduction in blood pressure and a significant increase in mortality in rats injected with doses of 30 and 60 µg/kg within 1 h. Successively, to better investigate the mechanisms underlying the inflammatory effects induced by *Pelagia noctiluca* crude venom injection, we showed the beneficial effects of tempol, a stable free nitroxide radical that works as an intracellular scavenger of superoxide anions and other free radical species. The reaction of tempol with superoxide anion to form hydrogen peroxide accounts for its “SOD mimetic” action. Tempol inhibits the catalytic action of transition metal irons and, hence, attenuates the formation of hydroxyl radicals [46]. All of these findings supported the view that tempol attenuated the degree of inflammation and oxidative stress caused by crude venom in rats, demonstrating a significant reduction of all inflammatory parameters measured in this study. Accordingly, we showed that crude venom injection increased organ dysfunction and injury, increasing blood parameters, such as levels of AST, ALT, bilirubin and alkaline phosphatase. In addition, high concentrations of lipase, amylase and creatinine, indicated the degree of organs dysfunction. In rats treated with tempol, a decrease in all blood parameters was observed as well as a significant reduction in the pathophysiology events related to inflammation. A histological resolution of organ damage following administration of tempol was also highlighted in lung and intestine by haematoxylin-eosin staining. Indeed, the degree of histoarchitectural modifications in these tissues significantly decreased after treatment with tempol. A recent evidence suggests that the activation of NF-κB p65 may be also under oxidant/antioxidant balance control [47]. Such hypothesis is based primarily on the observation that low doses of peroxides, including H_2_O_2_ and tert-butyl hydroperoxide, induce NF-κB p65 activation, whereas some antioxidants prevent it. Several studies have clearly demonstrated that tempol reduces the activation of the transcription factor NF-κB p65 [48,49]. We here report for the first time that *Pelagia noctiluca* crude venom caused a significant increase in the nuclear translocation of p65 in lung and intestine tissues 6 h after treatment, whereas treatment with tempol significantly reduced this activation. Moreover, we also demonstrated that tempol inhibited IκB-α degradation. The observed effect of tempol on NF-κB p65 activation is in agreement with previous studies [50]. NF-κB p65 plays a central role in the regulation of many genes responsible for the generation of mediators or proteins in inflammation. These include the genes for TNF-α, IL-1β, iNOS, and COX2. In this study, we showed that the expression of COX2 is increased in both lung and intestine of crude venom-injected rats, compared to the control group. Treatment with tempol significantly reduced the levels of COX2 in both organs. During inflammation initiation, circulating leukocytes must at first be able to adhere selectively and efficiently to vascular endothelium. This process is facilitated by induction of vascular cell adhesion molecules on the inflamed endothelium, such as vascular cell adhesion molecule VCAM-1, ICAM-1, E-selectin [51]. In this study, we observed that, 6 h after administration, crude venom induced the expression of P-Selectin in the endothelium of small vessels and up-regulated the surface expression of ICAM-1 and P-Selectin on endothelial cells in lung and intestine. In contrast, the expression of P-Selectin and ICAM-1 in lung and intestine was significantly lower than that one observed in tempol-treated rats. In this regard, we also showed a significant decrease in MPO activity, a marker of PMN accumulation, in animals injected with *Pelagia noctiluca* crude venom and treated with tempol. Both ROS [52,53] and NO have a role in the pathophysiology of crude venom-induced shock and inflammation. These species are cytotoxic agents, inducing lipid peroxidation and other cellular oxidative stress by cross linking proteins, lipids, and nucleic acids, which then cause cellular dysfunction, damage and eventually death [20,54]. Accordingly, in this study we demonstrated that tempol treatment caused a significant reduction in MDA levels, as an indicator of lipid peroxidation, in injured lung and intestine. In addition, we also demonstrated that tempol attenuated the expression of iNOS and plasma NO levels, evaluated as NO_2_/NO_3_. This inhibitory effect may be related to the already known inhibitory effect of tempol, on the activation of the transcription nuclear factor kappa B. Peroxynitrite, a cytotoxic oxidant species formed from the reaction of NO and superoxide, may mediate part of the oxidative injury associated with simultaneous production of NO and oxyradicals. Although peroxynitrite may play a role in normal cellular processes, excessive amounts of peroxynitrite will cause injury or death in a variety of cell types including leukocytes, neutrophils, endothelial cells and nerve cells. Nitrotyrosine formation, along with its detection by immunostaining, was initially proposed as a relatively specific marker for the detection of the endogenous formation of peroxynitrite. Moreover, an increased nitrotyrosine staining is considered as an indication of increased nitrosative stress. Thus, by immunohistochemical localization, we observed an increase in nitrotyrosine staining in lung and intestine obtained from crude venom-injured rats, while an improvement was due to tempol administration. A novel pathway of inflammation, governed by the nuclear enzyme PARP has been proposed in relation to hydroxyl radical and peroxynitrite-induced DNA single strand breakage [20]. Once PARP detects a single strand breakage, it binds to the DNA, and, after a structural change, begins the synthesis of a poly (ADP-ribose) chain (PAR) as a signal for the other DNA-repairing enzymes. We here demonstrated that tempol attenuates the increase in PARP activity in both lung and intestine of crude venom-injected rats. Thus, we proposed that the anti-inflammatory effects of tempol may be at least in part due to the prevention of PARP activation. Oxidative stress is involved in cell death in response to a variety of signals and pathophysiological conditions [48,49]. Therefore, we also identified pro-apoptotic transcriptional changes, including up-regulation of pro-apoptotic Bax and down-regulation of anti-apoptotic Bcl-2. In this study, we reported for the first time that tempol treatment in acute inflammation reduces apoptotic cell death after crude venom administration, suggesting that protection from apoptosis may be a prerequisite for anti-inflammatory approaches. In particular, we demonstrated that treatment with tempol lowers the signal for Bax in treated groups, when compared with lung and intestine sections of crude venom-treated rats, while, on the contrary, the signal was much more expressed for Bcl-2 in tempol-treated than in vehicle-treated rats. This meant that tempol had prevented the loss of the anti-apoptotic way by inhibiting NF-κB p65 and reduced the proapoptotic pathway activation with a mechanism still to be discovered.

## 4. Experimental Section

### 4.1. Animals

The study was carried out on Sprague-Dawley male rats (200–230 g, Harlan, Nossan, Italy). Food and water were available *ad libitum*. The study was approved by the University of Messina Review Board for the care of animals. Animal care was in compliance with Italian regulations on protection of animals used for experimental and other scientific purposes (D.M.116192) as well as with the EEC regulations (O.J. of E.C. L 358/1 12/18/1986).

### 4.2. Nematocysts Isolation and Crude Venom Extraction

Briefly, specimens of *Pelagia noctiluca* were collected in the Strait of Messina. The oral arms were excised and submerged in distilled water for 2 h at 4 °C. After a complete detachment of the epidermis the tissue was removed from the suspension containing both epidermis and undischarged nematocysts deriving from the osmotic rupture of nematocytes. The nematocysts suspension was repeatedly washed in cold distilled water and filtered through plankton nets (100, 60, and 40 μm mesh, respectively) to remove most of the tissue debris, and then centrifuged at 4 °C (ALC PK 120R, 4000× *g* for 5 min). The isolated nematocysts were classified as holotrichous isorhizas according to [55]. Then, the suspension was frozen at −20 °C until use. Before each experiment, the nematocysts were defrosted, filtered, and washed again in distilled water. This clean suspension of nematocysts was re-suspended in 0.01 M phosphate buffer containing 0.9% NaCl (pH 7.4; osmotic pressure = 300 mOsm/kg_H2O_). Crude venom was extracted by sonication on ice (Sonoplus, 70 mHz, 30 times, 20 s) of a population of isolated nematocysts (90 nematocysts/μL) and the crude extract was then separated from crushed capsules by refrigerated centrifugation at 4000× *g* for 10 min. The obtained venom was kept at 4 °C until use or stored at −20 °C or −80 °C, unless otherwise stated. Aliquots of crude venom were employed to measure the protein content by Bio-Rad Protein Assay (BioRad, Richmond, CA, USA), in agreement with previous studies [18].

### 4.3. Experimental Groups

The experiment was divided into two steps: in the first one, we preliminary evaluated the toxic effect of crude venom on blood pressure and mortality. For this purpose, rats (*n* = 25 rats/group) were randomly allocated into the following groups:

- Vehicle: group treated with saline alone. (*n* = 25 rats/group)
- Crude venom 6 μg/kg + Vehicle (saline): group intravenously injected with crude venom at the dose of 6 μg/kg. (*n* = 25 rats/group)
- Crude venom 30 μg/kg + Vehicle (saline): group intravenously injected with crude venom at the dose of 30 μg/kg. (*n* = 25 rats/group)
- Crude venom 60 μg/kg + Vehicle (saline): group intravenously injected with crude venom at the dose of 60 μg/kg. (*n* = 25 rats/group)

As a second step, to confirm the inflammatory effects caused by crude venom injection, we planned to treat crude venom-injected rats with an excellent antioxidant such as tempol. With this aim, the dose of 30 μg/kg of *Pelagia noctiluca* crude venom was used. Rats were randomly allocated into the following groups:

- Vehicle: group treated only with saline. (*n* = 25 rats/group)
- Tempol: group treated with tempol alone (100 mg/kg i.v. dissolved in saline). (*n* = 25 rats/group)
- Crude venom 30 μg/kg + Vehicle (saline): group injected by crude venom at the dose of 30 μg/kg. (*n* = 25 rats/group)
- Crude venom 30 μg/kg + Tempol: group injected by crude venom at the dose of 30 μg/kg and treated with tempol (100 mg/kg i.v. dissolved in saline) 30 min and 1 h after crude venom injection. (*n* = 25 rats/group)

The choice of this dose of tempol was based on our previous studies [56].

Six hours after administration of crude venom, rats were sacrificed and the following organs were taken: intestine, kidney, liver, lung. Blood samples, were centrifuged (1610× *g* for 3 min at room temperature) to separate plasma successively used for analysis of nitrites and nitrates, amylase, lipase, creatinine, bilirubin, AST, ALT and alkaline phosphatase levels.

### 4.4. Blood Pressure Measurement

Rats were anaesthetized by sodium pentobarbital (45 mg/kg i.p.). Following anaesthesia, catheters were placed in the carotid artery and jugular vein. The mean arterial blood pressure (MABP) was continuously recorded by a Maclab A/D converter (Ugo Basile Biological Research Apparatus, Varese, Italy), displayed and stored on a Macintosh personal computer. A rectal thermometer was inserted, and the rats were kept at a body temperature of 37–38 °C by a homeothermic blanket. The left jugular vein was cannulated to allow administration of further anesthetic and crude venom at three different doses 6, 30 and 60 µg/kg.

### 4.5. Myeloperoxidase Activity

Myeloperoxidase activity, which is an index of polymorphonuclear (PMN) leukocyte accumulation, was determined as previously described [57]. Intestine and lung tissues, collected 6 h after crude venom injection, were homogenized in a solution containing 0.5% hexa decyl-trimethylammoniumbromide dissolved in 10 mM potassium phosphate buffer (pH 7) and centrifuged for 30 min at 20,000× *g* at 4 °C. An aliquot of the supernatant was allowed to react with a solution of tetra-methyl-benzidine (1.6 mM) and 0.1 mM H_2_O_2_. The rate of change in absorbance was measured with a spectrophotometer at 650 nm. Myeloperoxidase activity was defined as the quantity of enzyme degrading 1 mmol of peroxide 1 min at 37 °C, and was expressed in units per gram weight of wet tissue.

### 4.6. Malondialdehyde (MDA) Measurement

Levels of MDA in the intestine and lung tissue were determined as an index of lipid peroxidation. Six hours after injection of crude venom, the animals were euthanized and the organs were removed with a scalpel. Each piece of tissue (100 mg) was homogenized in 1.15% KCl solution (1 mL per 250 mg of tissue). An aliquot (100 mL) of the homogenate was added to a reaction mixture containing 200 mL of 8.1% SDS, 1.5 mL of 20% acetic acid (pH 3.5), 1.5 mL of 0.8% thiobarbituric acid and 700 mL distilled water. Samples were then boiled for 1 h at 95 °C and centrifuged at 3000× *g* for 10 min. The absorbance of the supernatant was measured by spectrophotometry at 650 nm.

### 4.7. Measurement of Nitrite/Nitrate

Nitrite/nitrate (NO_2_^−^/NO_3_^−^) production, an indicator of NO synthesis, was measured in plasma collected 6 h after vehicle or crude venom administration. Plasma was incubated with nitrate reductase (0.1 U·mL^−1^), nicotinamide adenine dinucleotide phosphate (1 mM) and flavin adenine dinucleotide (50 mM) at 37 °C for 15 min, followed by further incubation with lactate dehydrogenase (100 U·mL^−1^) and sodium pyruvate (10 mM) for 5 min. Nitrite concentration in the samples was measured by Griess reaction, by adding 100 mL of Griess reagent [0.1% (*w*/*v*) naphthylethylenediamide dihydrochloride in H2O and 1% (*w*/*v*) sulphanilamide in 5% (*v*/*v*) H_2_PO_4_; vol. 1:1 to the 100 µL sample]. The optical density at 550 nm (OD550) was measured using an ELISA microplate reader (SLT-Lab Instruments, Salzburg, Austria). Nitrate concentrations were calculated by comparison with OD550 of standard solutions of sodium nitrate prepared in saline solution.

### 4.8. Histological Examination

For histopathological examination, the animals were euthanized 6 h following the intravenously injection of crude venom and tissues (intestine and lung) were removed with a scalpel. The tissue slices were fixed in Dietric solution (14.25% ethanol, 1.85% formaldehyde, 1% acetic acid) for 1 week at room-temperature, dehydrated by graded ethanol and embedded in Paraplast (Sherwood Medical, Sherwood, OR, USA). Section (thickness 7 μm) were deparaffinized with xylene, stained with Haematoxylin/Eosin (H&E), studied using light microscopy (Dialux 22 Leitz; Leica Microsystems SpA, Milan, Italy). Sections of each type of tissue were evaluated by an experienced histopathologist, without knowledge of the treatments. The following morphological criteria were used for scoring lung injury: 0, normal lung; grade 1, minimal oedema or infiltration of alveolar or bronchiolar walls; grade 2, moderate oedema and inflammatory cell infiltration without obvious damage to lung architecture; and grade 3, severe inflammatory cell infiltration with obvious damage to lung architecture. The following morphological criteria were used for intestine injury: 0, no damage; 1 (mild), focal epithelial oedema and necrosis; 2 (moderate), diffuse swelling and necrosis of the villi; 3 (severe), necrosis with the presence of neutrophil infiltrate in the submucosa; and 4 (highly severe), widespread necrosis with massive neutrophil infiltrate and haemorrhage.

### 4.9. Immunohistochemical Localization of iNOS, Nitrotyrosine, ICAM-1, P-Selectin and Poly ADP-Ribose (PAR)

Tissues, taken 6 h after crude venom injection, were fixed in 10% (*w*/*v*) PBS-buffered formaldehyde, and 8 mm sections were prepared from paraffin-embedded tissues. After de-paraffinization, endogenous peroxidase was quenched with 0.3% (*v*/*v*) hydrogen peroxide in 60% (*v*/*v*) methanol for 30 min. The sections were permeabilized with 0.1% (*w*/*v*) Triton X-100 in PBS for 20 min. Non-specific adsorption was minimized by incubating the section in 2% (*v*/*v*) normal goat serum in PBS for 20 min. Endogenous biotin or avidin binding sites were blocked by sequential incubation for 15 min with biotin and avidin (Santa Cruz, Milan, Italy) respectively. Sections were incubated overnight with the following antibodies: rabbit antinitrotyrosine (1:500 in PBS, *w*/*v*); anti-iNOS (1:500 in PBS, *w*/*v*); anti-PAR (1:500 in PBS, *v*/*v*); anti-ICAM-1 antibody (1:500 in PBS, *w*/*v*); anti-P-selectin (1:500 in PBS, *v*/*v*). Sections were washed with PBS, and incubated with secondary antibody. Specific labelling was detected with a biotin-conjugated goat anti-rabbit IgG and avidin-biotin peroxidase complex. The counter stain was developed with diaminobenzidine (brown) and nuclear fast red (red background). To confirm that the immunoreactions for the nitrotyrosine were specific, sections were incubated with primary antibody (anti-nitrotyrosine) in presence of excess nitrotyrosine (10 mM). Similarly, to confirm the binding specificity for iNOS, P-Selectin, ICAM-1 or PAR, some sections were incubated, alternatively, with primary or secondary antibody alone. Under these conditions, there was no positive staining indicating an effective immunoreaction. Immunocyto-chemistry photographs (*N* = 5) were assessed by densitometry by using Imaging Densitometer (AxioVision, Zeiss, Milan, Italy) and a computer program (AxioVision).

### 4.10. Western Blot Analysis for IκB-α, NF-κB p65, iNOS, COX2, Bax, and Bcl-2

Cytosolic and nuclear extracts were prepared as described previously [58] with slight modifications. Briefly, tissues from each rat were suspended in extraction buffer A (10 mM Hepes, 10 mM KCl, 0.1 mM EDTA, 0.1 mM EGTA, 1 mM DTT, 0.5 mM PMSF, 3 μg/mL pepstatin A, 2 μg/mL leupeptin, 15 μg/mL Trypsin inhibitor, 40 μM Benzamidine), homogenized at the highest setting for 2 min, and centrifuged at 13,000× *g* for 3 min at 4 °C. Supernatants were collected as the cytosolic fraction. The pellets containing nuclei were resuspended in buffer B (20 mM Hepes, 1.5 mM MgCl_2_, 0.4 M NaCl, 1 mM EGTA, 1 mM EDTA, 1 mM DTT, 0.5 mM PMSF, 3 μg/mL pepstatin A, 2 μg/mL leupeptin, 15 μg/mL Trypsin inhibitor, 40 μM Benzamidine, NONIDET P40 1%, Glycerol 20%). After centrifugation 10 min at 13,000× *g* at 4 °C, the supernatants were collected as nuclear extracts and then stored at −80 °C for further analysis. The levels of IκB-α, iNOS, COX2, Bax and Bcl-2 were quantified in cytosolic fraction from the tissue collected 6 h after crude venom administration, while NF-κB p65 levels were quantified in nuclear fraction. The blotting membranes were blocked with 1 PBS, 5% (*w*/*v*) nonfat dried milk (PM) for 1 h at room temperature and subsequently probed with specific Abs IκB-α (1:1000; Santa Cruz Biotechnology, DBA, Milan, Italy), or anti-Bax (1:500; Santa Cruz Biotechnology), or anti-Bcl-2 (1:500; Santa Cruz Biotechnology), or anti-iNOS (1:1000; Transduction) or anti-NF-κB p65 (1:1000; Santa Cruz Biotechnology), or anti-COX2 (1:1000; Santa Cruz Biotechnology) in 1 PBS, 5% *w*/*v* nonfat dried milk, 0.1% Tween-20 (PMT) at 4 °C, overnight. Membranes were incubated with peroxidase-conjugated bovine antimouse IgG secondary antibody or peroxidase-conjugated goat anti-rabbit IgG (1:2000; Jackson ImmunoResearch, West Grove, PA, USA) for 1 h at room temperature. To ascertain that blots were loaded with equal amounts of proteic lysates, they were also incubated in the presence of the antibody against β-actin protein (1:10,000 Sigma-Aldrich Corp., Milan, Italy). The relative expression of the protein bands of IκB-α (37 kDa), NF-κB p65 (65 kDa), Bax (23 kDa), Bcl-2 (29 kDa) iNOS (130 kDa), COX2 (72 kDa) was quantified by densitometric scanning of the X-ray films with GS-700 Imaging Densitometer (GS-700, Bio-Rad Laboratories, Milan, Italy) and a computer program (Molecular Analyst, IBM), and standardized for densitometric analysis to β-actin protein levels.

### 4.11. Quantification of Organ Function and Injury

Blood samples, taken 6 h after crude venom or vehicle injection, were centrifuged (1610× *g* for 3 min at room temperature) to separate plasma. All plasma samples were analyzed within 24 h in a veterinary clinical laboratory using standard laboratory techniques. The following enzymes were measured in plasma as indicators of multiple organ injury/dysfunction: (i) amylase and lipase as indicators of pancreatic injury; (ii) alkaline phosphatase, aspartate aminotransferase (AST, a non-specific marker for hepatic injury), alanine aminotransferase (ALT, a specific marker for hepatic parenchymal injury) and bilirubin as indicators of liver injury; (iii) creatinine, as an indicator of reduced glomerular filtration rate, and hence, renal failure.

### 4.12. Materials

Unless otherwise stated, all compounds used in this study were purchased from Sigma-Aldrich Company Ltd. (Poole, Dorset, UK). All solutions used for *in vivo* infusions were prepared using nonpyrogenic saline (0.9% *w*/*v* NaCl; Baxter Healthcare Ltd., Thetford, Norfolk, UK).

### 4.13. Statistical Analysis

All values in the figures and text are expressed as mean values ± standard error of the mean (SEM) of n observations. For *in vivo* studies, n represents the number of animals studied. In histology or immunohistochemistry experiments, figures are representative of at least three experiments performed on different experimental days. The results were analyzed by one-way ANOVA followed by a Bonferroni *post hoc* test for multiple comparisons. A *p*-value less than 0.05 was considered significant.

## 5. Conclusions

As reported in our previous papers [6,17,45], crude venom extracted from nematocysts of *Pelagia noctiluca* exerts both hemolytic and cytotoxic action at membrane level, displaying a pore formation or an oxidative effect, depending on the dose used and on the target chosen to test the venom. In addition to these reports, the present results show an oxidative effect elicited *in vivo*, reinforcing the hypothesis that *Pelagia noctiluca* venom acts via oxidative stress performed at cellular, tissue and systemic levels. Moreover, by using for the first time an *in vivo* model, we also demonstrate the capability of the venom, injected in rats, to induce apoptosis and inflammatory response. In this regard, the onset of the inflammatory response was associated with plasma concentrations of nitric oxide (NO) and ROS, maximal cellular infiltration, cyclooxygenase activity as well as lipid peroxidation. Finally, to better understand whether *Pelagia noctiluca* crude venom could have an important role in systemic inflammation, we proposed that small molecules, such as tempol, which permeate biological membranes and function as intracellular radical scavengers, may be useful in treating conditions associated with local or systemic inflammation [59].

Taken together, our findings contribute to the understanding of the mechanism of action of *Pelagia noctiluca* crude venom and open the way to further studies needed to support drug development from marine products.
